# The reliability and validity of DSM 5 diagnostic criteria for neurocognitive disorder and relationship with plasma neurofilament light in a down syndrome population

**DOI:** 10.1038/s41598-021-92887-5

**Published:** 2021-06-29

**Authors:** Sarah E. Pape, Tamara al Janabi, Nicholas J. Ashton, Abdul Hye, Rory Sheehan, Paul Gallagher, Bernice Knight, Anne-Marije Prins, Ken Courtenay, Vesna Jordanova, Bini Thomas, Nagarajan Perumal, Craig Forbes, Angela Hassiotis, Andre Strydom

**Affiliations:** 1 Institute of Psychiatry, Psychology, and Neuroscience, King’s College London, London, UK; 2 South London and Maudsley NHS Foundation Trust, London, UK; 3 Division of Psychiatry, University College London, London, UK; 4 NIHR Biomedical Research Centre for Mental Health and Biomedical Research Unit for Dementia, London, UK; 5 Department of Psychiatry and Neurochemistry, Sahlgrenska Academy at the University of Gothenburg, Mölndal, Sweden; 6 Wallenberg Centre for Molecular and Translational Medicine, University of Gothenburg, Gothenburg, Sweden; 7 Centre for Developmental Neurobiology and MRC Centre for Neurodevelopmental Disorders, King’s College London, London, UK; 8 North East London NHS Foundation Trust, London, UK; 9 Barnet Enfield and Haringey Mental Health NHS Trust, London, UK; 10 East London NHS Foundation Trust, London, UK; 11 Camden and Islington Mental Health Foundation Trust, London, UK; 12 Lancashire and South Cumbria, NHS Foundation Trust, Lancashire, UK; 13 Parnassia Psychiatric Institute, The Hague, The Netherlands; 14 Southern Health NHS Foundation Trust, London, UK; 15 Department of Forensic and Neurodevelopmental Sciences, Institute of Psychiatry, Psychology, and Neuroscience, King’s College London, London, SE5 8AF England

**Keywords:** Biomarkers, Biomarkers, Translational research, Dementia, Neurodegeneration, Neurodevelopmental disorders, Diagnosis, Dementia, Neurodegeneration, Neurodegenerative diseases

## Abstract

The validity of dementia diagnostic criteria depends on their ability to distinguish dementia symptoms from pre-existing cognitive impairments. The study aimed to assess inter-rater reliability and concurrent validity of DSM-5 criteria for neurocognitive disorder in Down syndrome. The utility of mild neurocognitive disorder as a distinct diagnostic category, and the association between clinical symptoms and neurodegenerative changes represented by the plasma biomarker neurofilament light were also examined. 165 adults with Down syndrome were included. Two clinicians independently applied clinical judgement, DSM-IV, ICD-10 and DSM-5 criteria for dementia (or neurocognitive disorder) to each case. Inter-rater reliability and concurrent validity were analysed using the kappa statistic. Plasma neurofilament light concentrations were measured for 55 participants as a marker of neurodegeneration and between group comparisons calculated. All diagnostic criteria showed good inter-rater reliability apart from mild neurocognitive disorder which was moderate (k = 0.494). DSM- 5 criteria had substantial concurrence with clinical judgement (k = 0.855). When compared to the no neurocognitive disorder group, average neurofilament light concentrations were higher in both the mild and major neurocognitive disorder groups. DSM-5 neurocognitive disorder criteria can be used reliably in a Down syndrome population and has higher concurrence with clinical judgement than the older DSM-IV and ICD-10 criteria. Whilst the inter-rater reliability of the mild neurocognitive disorder criteria was modest, it does appear to identify people in an early stage of dementia with underlying neurodegenerative changes, represented by higher average NfL levels.

## Introduction

The fifth edition of the diagnostic and statistical manual of mental disorders (DSM-5) reconceptualises dementia as part of a wider “neurocognitive disorder” (NCD), which is separated into mild and major subtypes^1^. The major subtype closely corresponds to previous definitions of dementia. The mild NCD category aims to identify individuals in an earlier stage of disease but there remains a lack of consensus in how it is applied and its validity is largely unknown^2,3^.

In order to be a valid and clinically useful concept, the diagnostic category of mild NCD needs to pick up early symptoms of dementia and be able to distinguish these from pre-morbid cognitive impairments^4^. Individuals with Down syndrome (DS) have both an ultra-high risk of developing Alzheimer’s dementia (AD)—usually at an earlier age than the general population—and intellectual disability which may affect reliability of clinical diagnoses, especially in early dementia stages^5–7^. As a result, individuals with DS are an important patient group in which to test the validity of the NCD criteria and explore their clinical utility in the timely assessment and diagnosis of dementia.

There is no current consensus on the optimum diagnostic criteria to use in people with DS and presentation can vary between individuals^8^. In light of this, comprehensive assessments have been recommended in this population^9^ and clinical judgement has performed better than the DSM-IV or ICD-10 dementia diagnostic criteria in previous studies^10^. We have thus considered clinical judgement as the “gold standard” to compare the performance of DSM-5 criteria against.

Neurofilament light (NfL) is a fluid biomarker shown to increase in neurodegenerative disorders including AD^11,12^. It can now be reliably detected in the plasma and these measurements have been shown to correlate with changes in cerebrospinal fluid. This makes it attractive as a less-invasive clinical tool to help improve diagnosis in dementia^13^. NfL has been studied in the DS population and shown to positively correlate with dementia diagnosis^14,15^ with high sensitivity and specificity^16^. We therefore used NfL to consider the biological validity of NCD criteria as an indicator of how well the criteria reflect underlying disease processes and neurodegeneration.

This study compared the diagnostic category of NCD with dementia criteria from ICD-10 and DSM-IV as well as the correlation with clinical judgement in individuals with DS. The aims were (1) to assess the inter-rater reliability and concurrent validity of the DSM-5 criteria for NCD in a large-scale study of cognitive decline in DS; (2) to consider the utility of mild NCD as a separate diagnostic category in a population with a genetically driven risk for AD; and (3) to explore the associations between NCD and underlying neurodegenerative changes represented by the plasma biomarker NfL. It was hypothesised that if the DSM-5 NCD criteria are reflective of underlying neurodegeneration, higher levels of plasma NfL would be found in individuals classed as NCD compared to those with no NCD.

## Methods

### Ethical approval

Ethical approval for the LonDownS study was granted from the North West Wales Research Ethics Committee (13/WA/0194). Written informed consent was obtained from individuals with capacity to consent after a full explanation of the study. In the event that individuals lacked capacity to provide informed consent, a consultee signed a form on their behalf to indicate their decision regarding the individual’s inclusion based on their knowledge of the individual and his/her wishes. This is in accordance with the UK Mental Capacity Act 2005.

### Application of dementia/NCD diagnostic criteria

Data were collected as part of the LonDownS study of people with DS over the age of 16 living in England and Wales using a validated assessment. The full battery has been published elsewhere^17^ and includes a mixture of direct and informant measures of cognition and adaptive abilities as well as demographic and physical health data.

One-hundred and sixty-five participants were selected from the “older adult” LonDownS cohort (participants over the age of 35 years) by the study co-ordinator using stratified random sampling to ensure a mix of ages and clinical dementia status. Data from the “younger adult” cohort (participants below the age of 35 years) were not used in this study due to the low prevalence of dementia in people with DS before 35: the average age of dementia diagnosis in DS is currently around 55 years old^18^. Diagnosis of DS was confirmed genetically using saliva or blood samples. Pre-existing dementia diagnosis was based on the medical history provided during the assessment.

For each participant the co-ordinator created anonymised case histories containing basic demographic information, medical history, medication lists, significant life events and psychiatric symptoms taken from the Mini PAS-ADD (Psychiatric Assessment Schedule for Adults with Developmental Disabilities)^19^. Information related to dementia symptoms was provided using the Cambridge Examination for Mental Disorders of Older People with Down’s Syndrome and Others with Intellectual Disabilities (CAMDEX-DS), a structured informant interview designed to pick up changes in an individual’s level of functioning over time^20^ that has been validated in a DS population for use as part of a dementia diagnostic process^21,22^. Raters were not involved in the case selection process or preparation of case histories.

Fifty-three participants (32.1%) had existing clinical diagnoses of dementia, 69 (42%) were female, 151 (91.5%) were of white ethnicity, 41 (24.8%) were APOε4 carriers. Further demographic data is available in Table 1.

**Table 1:** Demographic data.

	Whole sample	NfL subset
Number of participants	165	54
**Sex**		
Male	96 (58%)	34 (63%)
Female	69 (42%)	20 (37%)
**Chromosomal analysis**		
Trisomy 21	151 (91.5%)	52 (96.3%)
Mosaic	4 (2.4%)	1 (1.9%)
Translocation	2 (1.2%)	1 (1.9%)
Unspecified	8 (4.8%)	0
**Age**		
36–45	41 (24.8%)	12 (22.2%)
46–55	80 (48.5%)	25 (46.3%)
56–65	34 (20.6%)	12 (22.2%)
> 66	10 (16.5%)	5 (9.3%)
**Ethnicity**		
White European	151 (91.5%)	50 (92.6%)
Other	14 (8.5%)	4 (7.4%)
**Level of intellectual disability**		
Mild	58 (35.2%)	21 (38.9%)
Moderate	77 (46.7%)	28 (51.9%)
Severe/profound	27 (16.4%)	5 (9.3%)
Unstated	3 (1.8%)	0
**Epilepsy**		
Present	38 (23.0%)	15 (27.8%)
Absent	116 (70.3%)	36 (66.7%)
Unknown	11 (6.7%)	3 (5.6%)
**APOE status**		
APOε4 carrier	41 (24.8%)	10 (18.5%)
Non APOε4 Carrier	124 (75.2%)	44 (81.5%)

The cross-sectional data for each participant were presented to two clinicians with experience working in intellectual disability and dementia. The clinicians were blinded to participant ID, personal information, existing dementia diagnoses and use of dementia medication (acetylcholinesterase inhibitors or memantine). Using the available assessment data, both clinicians independently applied DSM-IV, ICD-10 and DSM-5 diagnostic criteria for dementia (or NCD) to each case vignette. In addition, each made a clinical judgment of no dementia, cognitive concern, possible dementia or certain dementia. Possible dementia was used when criteria for a diagnosis of dementia were met but it was not possible to fully rule out another cause for the symptoms based on the information available. Cognitive concern indicated the presence of subtle cognitive decline that would require investigation but which was insufficient for a dementia diagnosis.

After completing the independent ratings the clinicians reached a consensus rating for each diagnostic criteria and for clinical judgment. If there was disagreement, a third rater was involved to reach consensus. For this analysis the clinical judgement categories “possible dementia” and “certain dementia” were combined into a single clinical “dementia” category. “No dementia” and “cognitive concern” were collapsed into a single “no dementia” category. Inter-rater reliability and concurrent validity were assessed using the kappa statistic. The analyses were performed independently based on the individual rater diagnoses and consensus diagnoses respectively.

### Plasma NfL analysis

Fifty-five (33%) of the participants also provided a blood sample which was analysed for plasma NfL using the HD-1 Single molecule array (Simoa; Quanterix; Lexington, MA) platform. Full details of the laboratory process for blood analysis are described in Strydom et al.^14^. One participant was removed following sample analysis due to confounding factors—the participant had recently suffered a cerebrovascular accident leading to highly elevated NfL levels not specifically related to AD pathology (482 ng/L). This left a total sample of 54 participants.

Plasma NfL levels were compared between participants classified as having no dementia or dementia/NCD for each diagnostic criterion. Further analysis compared NfL levels in no NCD, mild NCD and major NCD as per DSM-5 criteria, and between those with no dementia, cognitive concern, or dementia according to clinical judgement. For this comparison possible and definite dementia were collapsed into one category. NfL data underwent logarithmic transformation to allow for statistical analysis using parametric tests.

Statistical analysis was performed using IBM SPSS statistics, version 25. Between group comparisons were performed using independent two tailed t-tests. Further analysis using one-way analysis of variance was completed on the fully differentiated groups. Linear regression was used to include relevant covariates in secondary analysis. These were age, sex, level of intellectual disability, presence of epilepsy and APOε4 status. Significance was set at p ≤ 0.05.

To further explore the associations between DSM-5 NCD criteria and underlying neurodegenerative change we applied a theoretical NfL cut-off to the three groups (no NCD, mild NCD, major NCD). Different NfL cut-off levels have been suggested to differentiate between people with and without dementia. For example Lewczuk et al. suggested a level of 25.7 pg/mL^11^. Recent work at King’s College London derived an NfL cut off at 30.01 ng/L (99% CI) to distinguish between people with and without AD below the age of 65 years. This was shown to have value in DS, with all DS participants with dementia having NfL values above this^16^. Using this NfL level (30.01 ng/L) we split our sample into those with low levels of neurodegeneration and those with higher levels of neurodegeneration and compared the distribution of these individuals in each of the three NCD groups.

## Results

### Application of diagnostic criteria

As shown in Table 2, all diagnostic criteria had good inter-rater reliability, as did clinical judgement. The DSM-5 NCD criteria as a dichotomous variable (NCD or no NCD) had strong inter-rater reliability (k = 0.711), although with a lower inter-rater reliability than ICD-10 (k = 0.866) and slightly lower than clinical judgement (k = 0.723). When the mild and major NCD categories were separated, the inter-rater reliability remained strong for major NCD (k = 0.727) but was only moderate for mild NCD (k = 0.494).

**Table 2:** Kappa statistic for inter-rater reliability and concurrent validity of each diagnostic criteria and clinical judgement.

Measure	Kappa
**Inter-rater reliability**	
DSM-IV	0.654
ICD-10	0.866
DSM-5 (any NCD)	0.711
DSM-5 major NCD	0.727
DSM-5 mild NCD	0.494
Clinical judgment	0.723
**Concurrent validity**	
DSM-5 & DSM-IV	0.555
DSM-5 & ICD-10	0.511
DSM-5 & clinical judgment	0.855
DSM-IV & clinical judgment	0.555
ICD-10 & clinical judgment	0.535
DSM-IV & ICD-10	0.759

Concurrent validity analysis showed substantial concurrence of DSM-5 diagnosis with clinical judgement (k = 0.855), but only moderate concurrence of DSM-5 with ICD-10 or DSM-IV criteria. Similarly, the ICD-10 and DSM-IV dementia criteria showed a lower concurrence with clinical judgement (k = 0.535 and 0.555 respectively) but substantial concurrence with each other (k = 0.759).

### NfL analysis

The median NfL level across the 54 participants was 33.2 ng/L (range 10.7–136.9 ng/L). The mean age of these individuals was 52.5 years (range 39–72 years, SD 8.66). Thirty-four (63%) were male.

Average NfL concentrations were measured for each of the diagnostic criteria (see Table 3). Significant differences in NfL concentrations were observed between participants diagnosed as no NCD compared to NCD (p = 0.003), and between those diagnosed as dementia or no dementia based on clinical judgement (p = 0.02). Differences in NfL between no dementia and dementia groups were non-significant when using DSM-IV or ICD-10 criteria (p = 0.148 and 0.08 respectively) as shown in Table 3.

**Table 3:** Grouped analysis of average NfL levels per diagnostic criteria, comparing no dementia and dementia.

	DSM-IV		ICD-10		Clinical Judgement		DSM-5	
	Median NfL ng/L (range)	Mean NfL ng/L (S.D.)	Median NfL ng/L (range)	Mean NfL ng/L (S.D.)	Median NfL ng/L (range)	Mean NfL ng/L (S.D.)	Median NfL ng/L (range)	Mean NfL ng/L (S.D.)
No Dementia or NCD	31.3 (10.6 –112.6)	37.6 (25.4)	30.2 (10.7–136.9)	37.6 (28.3)	27.5 (10.7–81.8)	32.2 (19.8)	24.9 (10.7–79.1)	29.7 (18.1)
Dementia or any NCD	50.3 (11.1–136.9)	58.4(40.7)	48.3 (11.1–111.1)	53.9 (32.9)	41.4 (11.1–136.9)	52.6 (35.5)*	41.4 (11.1–136.9)	53.1 (34.5)**

*Between group significance p < 0.05.

**Between group significance p < 0.01.

Further analysis using one-way ANOVA was completed on the fully differentiated groups for NCD and clinical judgement: three groups for NCD (no NCD, mild NCD, major NCD), and three groups for clinical judgement (no dementia, cognitive concern, possible/certain dementia).

Participants with major NCD and dementia were older than those without. In the groups based on DSM-5 criteria, those with mild NCD and major NCD were significantly older than those classified as having no NCD (p = 0.012 and 0.007 respectively) however there was no significant difference between the mild and major NCD groups (p = 0.46) in terms of age. In the groups based on clinical judgement there was no significance age difference between those classed as having no dementia compared to those with cognitive concern (p = 0.123), but those classed as having dementia were older than those with no dementia (p = 0.007). There was no significant difference in age between the cognitive concern and dementia groups (p = 0.21).

**Figure 1:**
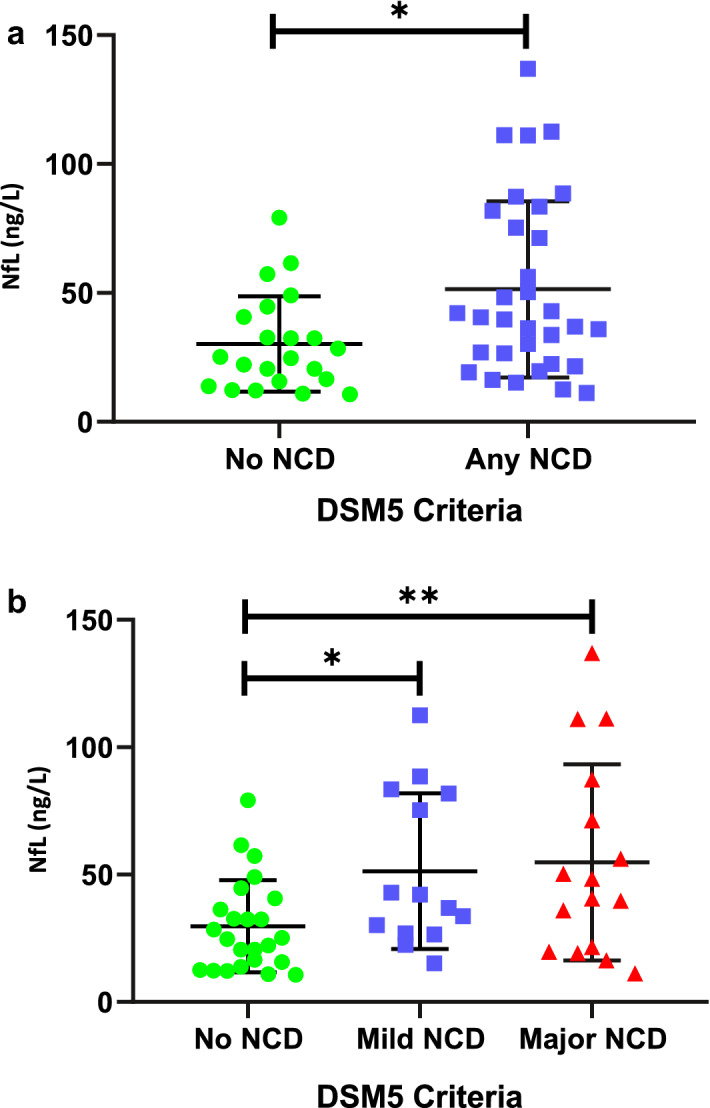
Histograms showing plasma NfL levels for participants based on DSM 5 NCD criteria, with mean and standard deviations. (**a**) Shows the distribution of individuals categorised as no NCD compared to those with either mild or major NCD. (**b**) Shows the distribution of NfL comparing those with no NCD, mild NCD and major NCD.

Mean NfL values were 29.7 (SD 18.1), 51.4 (SD 30.6) and 54.8 (SD 38.5) ng/L for no NCD, mild NCD and major NCD respectively. As displayed in Table 4, for the clinical judgement groups of no dementia, cognitive concern, and dementia, mean NfL values were 26.9 (SD 20.8), 38.3 (SD 17.3) and 52.6 (SD 35.5) ng/L. Significant and increasing NfL values were observed between the levels of each diagnostic criteria (between group differences p = 0.014 for NCD groups; p = 0.012 for clinical judgement). When compared to the no NCD group, average NfL concentrations were significantly higher in the mild NCD (p = 0.012) and major NCD (p = 0.025) groups. Similarly, when using clinical judgement there were significantly higher average NfL concentrations in the cognitive concern (p = 0.026) and possible/certain dementia (p = 0.006) groups compared to the no dementia group, see Fig. 1.

**Table 4:** Average NfL levels for individuals grouped according to DSM-5 criteria and for groups according to clinical judgement.

	N (%)	Mean age, years (SD)	Median NfL level ng/L (range)	Mean NfL ng/L (SD)
**NCD diagnosis**				
No NCD	24 (44)	48.3 (6.5)	24.9 (10.7 -79.1)	29.7 (18.1)
Mild NCD	14 (26)	54.5 (7.0)	39.6 (15.2–112.6)	51.4 (30.6)
Major NCD	16 (30)	56.9 (10.3)	44.4 (11.1 – 136.9)	54.8 (38.5)
**Clinical diagnosis**				
No Dementia	14 (26)	47.7 (6.8)	18.6 (10.7–79.1)	26.9 (20.8)
Cognitive Concern	12 (22)	51.8 (6.3)	32.5 (20.6 – 81.8)	38.3 (17.3)
Dementia	28 (52)	55.1 (9.5)	41.3 (11.1 – 136.9)	52.6 (35.5)

Average NfL concentrations were higher in the major NCD group compared to the mild NCD group and in the dementia group compared to the cognitive concern group, but these differences were not significant (p = 0.946 and p = 0.374 respectively).

Regression analysis was performed to allow inclusion of covariates with NfL as the dependent variable. Both DSM-5 criteria and clinical judgement had small but significant effects (DSM-5 group R^2^ 0.12, F7.17, sig 0.01; clinical judgement group R^2^ 0.12, F 7.30, sig 0.009). When age was added the overall model fit improved in both cases: DSM-5 group R^2^ 0.45, F21.2, sig 0.00; clinical judgement R^2^ 0.34, F9.67, sig 0.00. However, age showed significant suppressor effects on the categories and both DSM-5 group and clinical judgement lost significant in the model. No effects were found for sex, level of intellectual disability, APOε4 status or epilepsy.

An NfL cut off of 30.01 ng/L was applied to distinguish between people with lower and higher levels of neurodegeneration. The percentage of individuals with low (NfL < 30.01 ng/L) versus higher (NfL > 30.01 ng/L) levels of neurodegeneration was then calculated for each DSM-5 NCD category (no, mild or major NCD). For the 24 individuals categorised as no NCD, 42% (n = 10) had plasma NfL levels above the cut-off (> 30.01 ng/L). For mild NCD, 71% (n = 10) were above the cut-off, and for major NCD, 69% (n = 11) were above. Hence, the mild and major NCD diagnostic categories appear to identify more individuals with neurodegenerative changes that are, on average, more advanced than in those with no NCD. In addition, the mild NCD criteria may identify individuals with underlying neurodegenerative changes who clinically would not meet the criteria for major NCD.

## Discussion

People with DS have an ultra-high risk of developing AD dementia as they get older. However, there is significant variation between individuals regarding the age of onset and the clinical presentation. There can also be diagnostic uncertainty and delays in diagnosis due to the frequent presence of co-morbid intellectual disability and other health conditions that may impact on cognition in this population including thyroid disorders, sleep apnoea and depression^8^.

The high risk of AD coupled with the complexities in diagnosis make people with DS an ideal population in which to test the robustness of diagnostic criteria for dementia. To be clinically useful the criteria must be able to distinguish between pre-existing cognitive impairment and progressive changes related to dementia. We therefore explored the performance of the new DSM-5 NCD criteria in people with DS comparing this to existing criteria.

We found that NCD appears to have clinical utility when applied in a DS population. When viewed as a dichotomous variable (no NCD or NCD) we have shown it to have substantial inter-rater reliability, although major NCD criteria showed stronger inter-rater reliability than mild NCD. This may be because the mild NCD criteria reflect more subtle clinical changes, or because it is a newer diagnostic category that clinicians are less confident in applying. The DSM-5 NCD criteria also showed strong concurrent validity with clinical judgement, the current gold standard for diagnosing dementia in DS. This could indicate better reliability for use in clinical settings when compared to the older diagnostic criteria and may suggest that the NCD criteria could be used to help standardise diagnostic assessments.

To further explore the performance of NCD criteria in DS we analysed NfL concentrations in a subset of our sample. Whilst plasma NfL is a broad marker of neurodegeneration which increases with age, higher concentrations have been reliably reported in a variety of neurodegenerative disorders including AD^23,24^. Higher concentrations correspond to higher levels of neurodegeneration. In DS the development of AD is genetically driven and highly prevalent^5^. As such, even though NfL is a non-specific marker of neurodegeneration, increasing NfL levels can be assumed to be secondary to AD related changes in DS. Measuring the concentration of NfL can provide information about the progression of these changes, potentially before any clinical decline is apparent. In studies of autosomal dominant familial AD it has been proposed that NfL levels could predict age at symptom onset^25,26^. In DS, baseline NfL levels could potentially provide additional prognostic information for individuals if used in conjunction with tests of cognitive and adaptive functioning.

We showed that NfL levels were significantly increased on average in individuals diagnosed with NCD compared to those without NCD. The group identified as mild NCD had average NfL levels higher than in the non-NCD group but lower than in the major NCD group, despite there being no significant age differences between the mild and major NCD groups. The mild NCD criteria therefore appear to identify people with DS in the earlier stages of dementia, despite the lower inter-rater reliability. This adds weight to the assumption that the clinical presentation defined by mild NCD reflects neuropathological changes in the brain in the early stages of dementia. It also suggests a potential synergistic role for NfL and clinical criteria to be used to improve the dementia diagnostic process in DS.

Our results show a range of NfL values within diagnostic groups. The overlap in NfL levels observed between no NCD and mild NCD could be understood in the context of work in familial AD. Studies have found that NfL levels are increased even in pre-symptomatic mutation carriers^26^, and the rate of change of NfL rather than the absolute values may be more discriminatory during the early stages of AD in this population^27^. Therefore it may be expected that individuals with DS, who also develop a genetically driven form of AD, will show similar patterns in relation to NfL. In this case it is not surprising that there is variability in absolute values of NfL between individuals within each diagnostic group. In addition, we found a significant association between age and NfL. This is likely due to the strong correlation between age and NfL levels, which has been described previously^28^. It highlights the need for future work exploring correlations between age, rate of NfL change and clinical diagnostics in DS to help define clinical useful values to interpret NfL. It may be that serial measurements of NfL will be more useful than stand-alone values in the context of dementia in DS, or that age-specific cut-offs are required.

The strengths of our study include the large sample size for comparison of the diagnostic criteria in a wide range of individuals with DS using blinded and independent clinical ratings. In addition, data from a subset of these individuals has been linked to biomarkers (plasma NfL) linking recent research developments to clinical practice. This ultimately could lead the way towards more timely and precise diagnosis for individuals with DS and AD.

There are limitations in our study. Our sample size for biomarker analysis was relatively small which limited power. When selecting a cut-off for NfL we used information from studies that were not based purely on a DS population. As people with DS show early neuropathological changes^29^, it is likely that the cut-offs for markers of neurodegeneration will need to be adjusted if they are being considered for clinical use in people with DS.

This study shows that DSM-5 NCD criteria can be used reliably in a DS population despite the presence of premorbid cognitive impairments. Whilst the inter-rater reliability of the mild NCD criteria was only modest, it does appear to identify a distinct group of people with underlying pathological changes but with only limited cognitive and functional decline who could be considered to be in an early stage of dementia. Furthermore, the combined categories of NCD had better concurrent validity against clinical judgement and performed better at biomarker level than the older DSM-IV criteria.

By bridging the gap between clinical symptoms and lab-based biomarkers in dementia there is the potential to improve early detection of this disease. Our study considers how these tools can be considered synergistically, as a potential way to confirm clinical diagnoses and incorporate newer laboratory techniques whilst maintaining a focus on an individual’s symptoms and presentation. Whilst further work is needed to define how NfL concentrations can be applied in a clinical setting, there is promise that a combined approach can provide more accurate information about a person’s current needs and may also assist in predicting future disease progression and care planning. This is vital in neurodegenerative conditions such as dementia that require coordinated input from various agents as the disease progresses and may allow for resource demand to be more effectively estimated and allocated to best support individual needs.

## Data Availability

The datasets used and/or analysed during the current study are available from the corresponding author on reasonable request.

## Abbreviations

DS: Down syndrome
NfL: Neurofilament light
AD: Alzheimer’s dementia
NCD: Neurocognitive disorder
DSM IV: Diagnostic and statistical manual of mental disorders, 4th edition
DSM 5: Diagnostic and Statistical Manual of Mental Disorders, 5th edition
ICD 10: International Classification of Diseases, 10th revision

## References

[1] American Psychiatric Association (2013). Diagnostic and Statistical Manual of Mental Disorders.

[2] Stokin GB, Krell-Roesch J, Petersen RC, Geda YE (2015). Mild Neurocognitive disorder: An old wine in a new bottle. Harv. Rev. Psychiatry.

[3] Sachdev PS (2014). Classifying neurocognitive disorders: The DSM-5 approach. Nat. Rev. Neurol..

[4] Aylward EH, Burt DB, Thorpe LU, Lai F, Dalton A (1997). Diagnosis of dementia in individuals with intellectual disability. J. Intellect. Disabil. Res..

[5] Wiseman FK (2015). A genetic cause of Alzheimer disease: Mechanistic insights from Down syndrome. Nat. Rev. Neurosci..

[6] McCarron M (2017). A prospective 20-year longitudinal follow-up of dementia in persons with Down syndrome. J. Intellect. Disabil. Res..

[7] Firth NC (2018). Aging related cognitive changes associated with Alzheimer's disease in Down syndrome. Ann. Clin. Transl. Neurol..

[8] Ballard C, Mobley W, Hardy J, Williams G, Corbett A (2016). Dementia in Down's syndrome. Lancet Neurol..

[9] Lautarescu BA, Holland AJ, Zaman SH (2017). The early Presentation of dementia in people with Down syndrome: A systematic review of longitudinal studies. Neuropsychol. Rev..

[10] Sheehan R (2015). Dementia diagnostic criteria in Down syndrome. Int. J. Geriatr. Psychiatry.

[11] Lewczuk P (2018). Plasma neurofilament light as a potential biomarker of neurodegeneration in Alzheimer’s disease. Alzheimer's Res. Ther..

[12] Ashton NJ (2019). Increased plasma neurofilament light chain concentration correlates with severity of post-mortem neurofibrillary tangle pathology and neurodegeneration. Acta Neuropathol. Commun..

[13] Hampel H (2018). Blood-based biomarkers for Alzheimer disease: Mapping the road to the clinic. Nat. Rev. Neurol..

[14] Strydom A (2018). Neurofilament light as a blood biomarker for neurodegeneration in Down syndrome. Alzheimer's Res. Ther..

[15] Fortea J (2018). Plasma and CSF biomarkers for the diagnosis of Alzheimer's disease in adults with Down syndrome: A cross-sectional study. Lancet Neurol..

[16] Ashton NJ (2021). A multicentre validation study of the diagnostic value of plasma neurofilament light. Nat Commun..

[17] Startin C (2016). The LonDownS adult cognitive assessment to study cognitive abilities and decline in Down syndrome [version 1; referees: 2 approved]. Wellcome Open Res..

[18] Sinai A (2017). Predictors of age of diagnosis and survival of Alzheimer's disease in down syndrome. J. Alzheimer's Dis..

[19] Prosser H (1998). Reliability and validity of the Mini PAS-ADD for assessing psychiatric disorders in adults with intellectual disability. J. Intellect. Disabil. Res..

[20] Ball S, Holland T, Huppert FA, Treppner P, Dodd K (2006). The CAMDEX-DS: The Cambridge Examination for Mental Disorders of Older People with Down's Syndrome and Others with Intellectual Disabilities.

[21] Ball SL (2004). The modified CAMDEX informant interview is a valid and reliable tool for use in the diagnosis of dementia in adults with Down's syndrome. J. Intellect. Disabil. Res..

[22] Fonseca LM (2019). The validity and reliability of the CAMDEX-DS for assessing dementia in adults with Down syndrome in Brazil. Braz. J. Psychiatry.

[23] Gaetani L (2019). Neurofilament light chain as a biomarker in neurological disorders. J. Neurol. Neurosurg. Psychiatry.

[24] Mattsson N, Cullen NC, Andreasson U, Zetterberg H, Blennow K (2019). Association between longitudinal plasma neurofilament light and neurodegeneration in patients with Alzheimer disease. JAMA Neurol..

[25] Sánchez-Valle R (2018). Serum neurofilament light levels correlate with severity measures and neurodegeneration markers in autosomal dominant Alzheimer's disease. Alzheimer's Res. Ther..

[26] Weston PSJ (2017). Serum neurofilament light in familial Alzheimer disease: A marker of early neurodegeneration. Neurology.

[27] Preische O (2019). Serum neurofilament dynamics predicts neurodegeneration and clinical progression in presymptomatic Alzheimer's disease. Nat. Med..

[28] Khalil M (2020). Serum neurofilament light levels in normal aging and their association with morphologic brain changes. Nat. Commun..

[29] Mann DMA, Esiri MM (1989). The pattern of acquisition of plaques and tangles in the brains of patients under 50 years of age with Down's syndrome. J. Neurol. Sci..

